# Successful trans-apical aortic valve implantation for a high risk patient with aortic stenosis using a new second-generation TAVI device — J-Valve™ system

**DOI:** 10.1186/s13019-015-0207-z

**Published:** 2015-01-17

**Authors:** Jiahan Cheng, Miao Chen, Da Zhu, Ji Zhang, Jia Hu, Yingqiang Guo

**Affiliations:** 1 Department of Cardiovascular Surgery, West China Hospital, Sichuan University, Chengdu, Sichuan P.R. China; 2 West china school of Clinical Medicine, Sichuan University, Chengdu, China; 3 JC Medical Inc., Florida, CA USA

**Keywords:** Trans-catheter aortic valve implantation (TAVI), Severe aortic stenosis, J-Valve™ system, High risk patient, Second generation TAVI device

## Abstract

Transcatheter aortic valve implantation (TAVI) has evolved as a routine procedure to treat selected high-risk patients with severe aortic stenosis. The new J-Valve™ prosthesis is designed for antegrade transapical implantation, it is characterized by a porcine aortic prosthesis attaching to a self-expandable Nitinol stent. The key feature of the device are three U-shape anatomically oriented devices - “graspers” which could facilitate intuitive ‘self-positioning’ valve implantation. Hereby, we report a successful case of trans-apical TAVI in an elderly high-risk patient with severe aortic stenosis using J-Valve™ system.

## Background

Transcatheter aortic valve implantation (TAVI) has been recognized as a minimally invasive treatment option for patients with inoperable or high-risk symptomatic aortic stenosis [1]. The J-Valve™ system is a brand new second generation TAVI device featured by a porcine aortic prosthesis attaching to a self-expandable Nitinol stent and three U-shape anatomically oriented devices “graspers” encircling around the stent. This unique design could facilitate intuitive “self-positioning” valve implantation and provide both axial as well as radial fixation by embracing the native valve leaflets. We performed the first-in-man case of successfully TAVI operation for a high-risk patient with severe aortic stenosis using the J-Valve™ system through trans-apical approach.

## Case report

An 80 years old female was referred to our hospital due to severe aortic stenosis and recurrent episodes of decompensated left heart failure. She presented with a history of stroke 7 month ago with no obvious sequelae. Transthoracic echocardiogram (TTE) confirmed a severe aortic stenosis with a mean pressure gradient of 58 mmHg and aortic valve opening area 0.6 cm^2^. Extra-cardiac arterial disease involving carotid, intra-cranial and lower limb arteries was confirmed through vascular ultrasound and MRI angiogram. Preoperative CT angiogram revealed a heavily calcified aortic valve with aortic annulus diameter 25 mm (derived from annulus perimeter). After interdisciplinary assessment, the patient was scheduled for trans-apical aortic valve implantation because of a significantly increased risk for conventional surgery. (Logistic EuroSCORE = 28%). The informed consent was obtained after discussing all available treatment options. Ethic committee of our hospital also approved this procedure.

**Figure 1:**
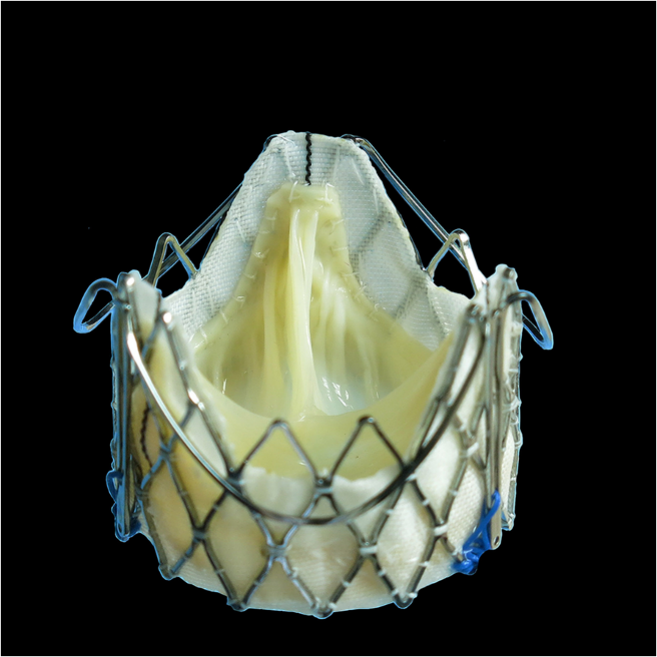
**The J-Valve™ system.** J-Valve™ is composed of a porcine aortic valve attaching to a self-expandable nitinol stent with three U-shape graspers encircling the valve stent.

**Figure 2:**
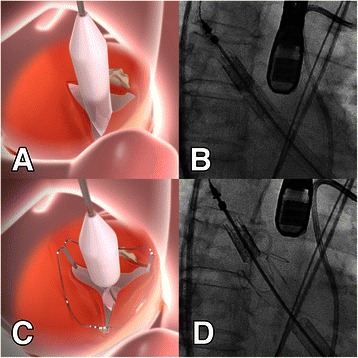
**The implantation process of J-Valve™ system (Part I).** Panel **A-B**: the delivery sheath was sent into the supra-annular plan through transapical approach. Panel **C-D**: Three graspers (small black arrow) were then totally released and pushed back gently into the aortic sinuses, angiogram was performed to ensure the correct position of the graspers.

**Figure 3:**
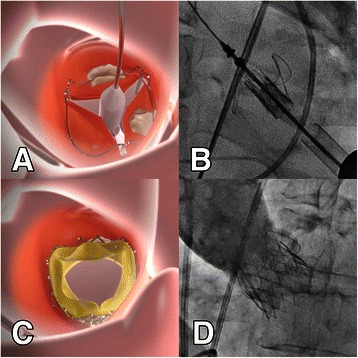
**The implantation process of J-Valve™ system (Part II).** Panel **A-B**: The valve was retrieved into the annular plan with the help of the locking device. Panel **C-D**: The valve was then deployed without rapid ventricular pacing, further angiogram confirmed good position of the valve while without obvious paravalvular leakage.

**Figure 4:**
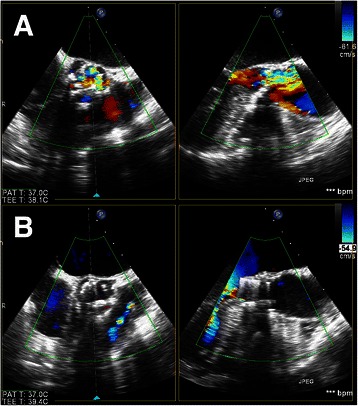
**Pre and post-operative TEE image of this patient.** Panel **A**: Preoperative TEE reveled a calcified aortic valve with severe stenosis. Panel **B**: Both short and long axis view of TEE confirmed a good valve position with only trivial grade para-valvular leakage.

The procedure was performed under general anesthesia in a fully equipped hybrid operating room. Prior to skin incision, a femoral ‘safety-net’ (percutaneous wires in the femoral vessels) was placed and a readily primed CPB machine was available in the room for immediate CPB if required. Temporary pacemaker was placed in the right ventricle through right internal jugular vein and then the standard apical access was obtained. A cranial left-anterior-oblique angulation was chosen by adjusting the angulation of the fluoroscopic system which could provide good visualization of each commissure of the aortic valve. The apical puncture was done with a super-stiff guide-wire placed in position. The standard apical aortic valvuloplasty was performed using a straight 24-mm balloon under rapid ventricular pacing (RVP). In parallel, a 27-mm J-Valve™ was crimped into the Ausper-AS delivery system (JC Medical Inc., Florida, California, USA) (Figure 1). The delivery system was bluntly inserted into the left ventricle and advanced under fluoroscopic guidance into a supra-annular position (Figure 2). In the next step, the three ‘U-shape graspers’ were totally released and care was taken to bring each grasper into the corresponding aortic sinus by gently pulling back the delivery system, thereby embracing the native leaflets (Figure 2). The correct position was identified on both fluoroscopy as well as trans-esophageal echocardiogram (TEE). Then the valve was pulled back gently into the annular plan with the guidance of the graspers and deployed without rapid ventricular beating (Figure 3). After retrieval of the delivery system, aortic root angiography revealed only trivial para-valvular leakage with patent coronaries and good valve stent position (Figure 3). TEE also confirmed a good valve position with only trivial grade para-valvular leakage (Figure 4).

After wound closure, the patient was transferred to the intensive care unit. Further postoperative course was uneventful. At discharge, TTE revealed an excellent valve function without any relevant incompetence and obvious paravalvular leakage. The maximum and mean transvalvular gradients were 18 and 11 mmHg measured by Doppler ultrasound. During the 3-month follow-up, this patient remained in good health, TTE confirmed an optimal valve position with no paravalvular leakage.

## Discussion

TAVI has evolved as a routine procedure for high risk patient with severe aortic stenosis. Medtronic CoreValve™ and Edward SAPIEN™ are two classic TAVI systems which have been widely used with accepted results [2,3]. However, these first-generation systems still have specific drawbacks such as highly technique requirements for precise valve positioning, relatively frequent requirement for pace-maker implantation as well as high risk for more than mild degree para-valvular leakage [2,3].

Next-generation transcatheter valves should overcome the known limitations of the currently available TAVI. In this case, we used the brand new J-Valve™ TAVI system. Sharing the similarity with other second-generation TAVI devices with anatomically oriented devices such as Jena-Valve™ and Medtronic Engager Valve™ [4,5], this system is characterized by a unique design U-shape graspers which could be totally released before the valve implantation facilitating intuitive ‘self-positioning’ valve implantation. This unique design could greatly simplify the implantation process and lead to more consistent results, because the operator does not need to actively judge for optimal valve position. Meanwhile, through embracing the native valve leaflets through three U shape graspers, J-Valve™ system has the advantage of achieving better anchoring, less risk for coronary obstruction from native cusp calcification, as well as less risk of conduction block (extra-axial fixation and subsequent less radial expansion forces).

Also different from other second-generation TAVI devices, U-Shape graspers of J-Valve™ system could increase the interface between aortic valve leaflets and valve prosthesis which would provide more anchorage force (axial fixation) after valve deployment. In addition, the unique two stage releasing design of this system (graspers totally release first and followed by the valve release) could also largely facilitate accurate adjusting the position of the graspers before valve deployment. Furthermore, compared with single arm feeler device of Jena-valve™, this U-shape grasper could also minimize the risk of native valve perforation.

## Conclusion

The new J-Valve™ prosthesis is a brand new second generation TAVI device. It is featured by three U-shape anatomical orientated devices - “graspers” which could largely facilitate intuitive ‘self-positioning’ valve implantation. As shown in this case, this system provide a potential valuable treatment option for high risk patients with severe aortic stenosis.

## Consent

Note: Written informed consent was obtained from the patient for publication of this case report and any accompanying images. A copy of the written consent is available for review by the Editor-in-Chief of this journal.

## Abbreviations

TAVI: Transcatheter aortic valve implantation
TTE: Transthoracic echocardiogram
TEE: Transesophageal echocardiogram
